# Annotations

**Published:** 1902-10-04

**Authors:** 


### Annotations.

### The Death of M. Zola.

While with the rest of the world we grieve over the sudden death of M. Zola, we must not let the manner of it pass without comment, serving as it does to emphasise and illustrate the danger which, although coming in a different form, always accompanies the practice of sleeping with closed windows. As in so much that Zola wrote, so in the manner of his death we have, concentrated upon one tragic page, what in ordinary circumstances is scattered over space and time. In the case of the great novelist death came suddenly by the hand of a poisonous gas, while in other cases it has worked slowly by means of a destructive microbe, but in both alike it has been the lack of ventilation which enabled the agent to do its work. That consumption is caused by a bacillus ; that this bacillus exists in the expectoration of those who suffer, even in slight degree, from the disease ; that when dried up and fluffed out of handkerchiefs in the form of light impalpable dust it impregnates the air ; that when this air is breathed and re-breathed by those who live in unventilated rooms it causes consumption ; and that consumption is still the deadliest disease we have amongst us, are now truisms. Yet we go on shutting our windows as if these things were merely fairy tales. In the newspaper accounts of M. Zola's death we are told that his window "naturally had to be closed at night," as if so doing were quite a matter of course, as indeed it is in ninety-nine houses out of a hundred. The English are always boasting that they are a cleanly people, and undoubtedly an upper-class Englishman does spend an inordinate amount of time in cleansing himself. As "Punch" has it, "'e's orful proud of 'is flesh, 'e is." But the average Englishman with his woollen shirts, which are only washed in tepid water, his cloth clothes which are never washed at all, his carpets which retain the dust of years, his stuffy woollen-covered furniture which lasts for generations, and with his beds which are hardly ever unpicked or stoved—is by no means the cleanly animal that he thinks himself. Still, even these things would not be so bad if John Bull would but ensure a free current of air all through his living rooms. But that is just what Mrs. John Bull will by no means allow. Fresh air is "smutty" and night air is "unhealthy," so the windows must be shut. What sort of night air could be more "unhealthy" than the fogs which cover the river Thames, and what air could be harsher than that of Hampstead in mid-winter? Yet all winter through there are sick people lying out on the verandahs at St. Thomas's Hospital and at the Mount Vernon Consumption Hospital, being brought round again to life by this "unhealthy" air after being nearly killed—by inches, it is true—by the very thing that killed M. Zola all at once, namely, the lack of open windows.

### Anti-scarlatinal Serum.

The announcement comes from Vienna that Dr. Moser has been able to produce an antitoxic serum for the treatment of scarlet fever, and that an experience of nearly 400 cases has proved it to be eminently successful. Let us not fall, however, into the easy error of accepting this statement as indicating any startling therapeutic advance. The main question to us in England is as to the influence which such a serum when discovered may have upon the progress of the disease, for while all sera have their disadvantages (so that the justifiability of employing them depends upon the gravity of the disease for the treatment of which they are proposed), the gravity of scarlet fever, whether we regard its prevalence or its fatality, has much diminished during recent years. The average case-mortality of scarlet fever in London had in the year 1900 come down to 2·6 per cent., so that the statement that the serum treatment had reduced the mortality in Vienna to 8 or 9 per cent. does not at first sight seem encouraging. We must remember, however, that the whole meaning of these figures hinges on the question of age, for, notwithstanding the low average case-mortality reached in London, scarlet fever is still a very fatal disease when it attacks young children, the case-mortality mounting up as high as 16·5 per cent. among boys and 10·1 per cent. among girls under one year of age, but rapidly dropping to about 1 per cent. among children between five and ten years of age. Hence, until one knows the ages of the children treated in Vienna, it is impossible to say what is the meaning to be attached to the figures which have been given.

### A Snarl from Birmingham.

A curious document has been issued by Mr. Arthur Chamberlain, as Chairman, and Mr. Acton as Hon. Secretary of the late Birmingham Consultative Medical and Surgical Institute, in which after giving a history of the initiation and subsequent abandonment of the movement it is stated that the work of the institution was brought to a standstill by the mere force of the Doctors' Trade Union. Mr. Arthur Chamberlain and those who are acting with him seem even yet to be under a strange misconception as to what is meant by "consulting advice." Nothing is simpler than to provide the working classes of Birmingham with consulting advice at a cheap rate ; the difficulty is to induce the said working classes to accept it when the "consultant" who is set up is one whom they do not care to consult. That the Birmingham Consultative Institution could obtain plenty of half-crown men who were willing to accept half-a-guinea went without saying. The difficulty lay in the astuteness of the Birmingham working man, who, curiously enough, did not seem to care to pay half-a guinea for the sort of advice which the Institute could offer them. What upset the Institute was that the working man found out that he could do far better in the open market. It is absurd to talk about medical trade unionism—many doctors only wish there was such a thing. What knocked the Birmingham Institute on the head was the apparent ignorance of its promoters as to the sort of "consulting advice" the working classes wanted, coupled with the fact that the Birmingham working man knew all the time that by showing his necessity and his inability to pay a full fee, he could any day obtain the real thing for the same fee as that for which the Institute offered him its wonderful "consultants."

